# Implication of creative potential in bipolar disorder: a study conducted with an artistic population

**DOI:** 10.1192/j.eurpsy.2026.11823

**Published:** 2026-07-31

**Authors:** H. Mezghani, N. Regaieg, F. Guermazi, F. Cherif, I. Feki, J. Masmoudi, I. Baati

**Affiliations:** Psychiatry A Department, Hedi Chaker University Hospital, Sfax, Tunisia

## Abstract

**Introduction:**

Significant creative resources facilitate the expression of feelings. However, could the intensity and instability of this emotional involvement become a source of emotional dysregulation and also constitute a factor in mood vulnerability?

**Objectives:**

The objective of our study is to study the associations between creative skills and bipolar traits among a sample of artistic students.

**Methods:**

Our study was cross-sectional, descriptive, and analytical, conducted among students at the Institute of Arts and Crafts in Sfax (Tunisia), from the beginning of February to the beginning of March 2025. Data collection was based on an information sheet containing sociodemographic, clinical, and two psychometric scales in their Arabic versions: the Epstein Creativity Competencies Inventory for Individuals (ECCI-i), measuring four dimensions of creativity (idea capture, questioning, broadening perspectives, influence of environment) and the Mood Disorder Questionnaire (MDQ), used for screening for bipolar disorder.

**Results:**

Sixty students participated in the study, divided into 23 men (38.3%) and 37 women (61.7%). The median age was 20 years with extremes of 19 and 22 years.

Creative skills among students were low in 3.3% of cases, medium in 23.3% of cases and high in 73.3% of cases.

Almost half of the students (45%) considered that their desire for artistic expression influences their social and family relationships.

Furthermore, 41.7% of students perceived creativity as a source of pressure related to the need to produce and perform. In addition, 45% of students relied on their creative process to manage and regulate their emotions.

Seventeen students (28.3%) had a positive MDQ score. The percentage of positive MDQ scores was significantly higher among students with high creative skills compared to those with low or medium creative skills (p = 0.025).

**Conclusions:**

Developed artistic expression skills appear to impact mood vulnerability. Conversely, creativity may also, in certain contexts, promote mental health. In this regard, it is important to study other factors that may modulate the risk of bipolar disorder.

**Disclosure of Interest:**

None Declared

